# Outer membrane engineering through lipid A modification allows modulation of *Burkholderia pseudomallei* membrane permeability and innate immune responsivity

**DOI:** 10.1128/iai.00602-25

**Published:** 2026-07-22

**Authors:** Thi Hai Au La, Ian A. McMillan, Prashant Dahal, Jourdan K. P. McMillan, Artur Muszyński, Christian Heiss, Liyanage Devthilini Fernando, Parastoo Azadi, Bradley R. Borlee, Michael H. Norris

**Affiliations:** 1 Pathogen Analysis and Translational Health Group, School of Life Sciences, University of Hawaiʻi at Mānoa 166868, Honolulu, Hawaii, USA; 2 Daniel K. Inouye Center for Microbial Oceanography: Research and Education, School of Ocean and Earth Science and Technology, University of Hawaiʻi at Mānoa 124316, Honolulu, Hawaii, USA; 3 Department of Tropical Medicine, Medical Microbiology, and Pharmacology, John A. Burns School of Medicine, University of Hawaiʻi at Mānoa 50677, Honolulu, Hawaii, USA; 4 Complex Carbohydrate Research Center, University of Georgia 123423, Athens, Georgia, USA; 5 Department of Microbiology, Immunology and Pathology, Infectious Disease Research Center, Colorado State University 164597, Fort Collins, Colorado, USA; Stanford University School of Medicine, Stanford, California, USA

**Keywords:** *Burkholderia pseudomallei*, melioidosis, lipid A, MALDI-TOF, lipopolysaccharide, intracellular pathogen, outer membrane vesicles, immune response

## Abstract

*Burkholderia pseudomallei* causes the tropical disease melioidosis. Host mortality primarily results from sepsis-related complications and associated cytokine release. Lipopolysaccharide (LPS), a major virulence factor and mediator of sepsis, is modified by *B. pseudomallei* in response to external stimuli. LpxO and PagL are lipid A-modifying proteins encoded by the *B. pseudomallei* genome to decrease recognition by host defenses. The contribution of *lpxO* and *pagL-*dependent lipid A modifications to outer membrane permeability and host response has not been clearly demonstrated. Mono-phosphorylated lipid A (MPLA) is known to maintain LPS-specific immunogenicity while reducing endotoxicity and has been used extensively as a vaccine adjuvant. Generation of MPLA can be mediated through LpxE. In this work, a panel of defined lipid A modification strains in *B. pseudomallei* was generated to study effects on bacterial physiology and cytokine expression in macrophages. Knockout of *lpxO* and *pagL* resulted in dehydroxylation and penta-acylation of lipid A, respectively, and reduced membrane permeability of *B. pseudomallei*. Expression of *lpxE* removed one phosphate group as measured by matrix-assisted laser desorption/ionization time-of-flight (MALDI-TOF) and caused increased membrane permeability. Outer membrane vesicles (OMVs) from the generated mutants were used to study the immunological effect of lipid A modifications on human monocyte-derived macrophages. Macrophages treated with OMVs isolated from *lpxE*-expressing strains showed significant increases in gene expression of proinflammatory cytokines tumor necrosis factor and interferon gamma compared to wild-type *B. pseudomallei* OMVs. These results show the importance of lipid A modifiers LpxO and PagL for *B. pseudomallei* physiology and highlight the immunostimulatory effect of *lpxE*-modified OMVs that could enhance OMV vaccine technology targeting melioidosis.

## INTRODUCTION

The exterior surface of a bacterial cell interacts with the environment and allows physiological adjustments for survival. The asymmetric outer membrane of gram-negative bacteria contains an outer leaflet comprised of the glycolipid lipopolysaccharide (LPS) that creates a barrier to external toxic compounds, including antibiotics (1, 2). The major components of LPS are the exterior O-antigen, core oligosaccharide, and conserved lipid A backbone. The distal O-antigen is highly variable and comprised of various repeating oligosaccharides directly linked to the core oligosaccharide (3–5). The core oligosaccharide often contains non-repeating heptose and hexose residues, which can be modified with phosphates, phosphoethanolamine, uronic acid, or other substituents, and is anchored through ketodeoxyoctonic acid (Kdo) residues directly linked to glucosamines that are the lipid A backbone (6–9). The prototypical lipid A structure has biphosphorylated glucosamines (GlcN_I_ and GlcN_II_) attached in a β(1→6) linkage and a total of six acyl chains (10). The resulting biphosphorylated hexa-acylated disaccharide is highly immunogenic to the human innate immune system via its recognition by the Toll-like receptor 4/myeloid differentiation factor 2 (TLR4/MD-2) immune complex (11–19). Structural modifications of the lipid A backbone can significantly alter the physiology and physical characteristics of gram-negative bacteria by altering recognition by the immune system, resistance to antimicrobial peptides, and membrane permeability (10, 20). Dephosphorylation and changes in acylation of the lipid A backbone are two strategies gram-negative bacteria use to modify immune signaling (11, 12, 21). Dephosphorylation of the lipid A backbone in *Helicobacter pylori* via LpxE and LpxF results in reduced TLR4 activation, allowing for gastric colonization (22). In *Salmonella enterica* serovar Typhimurium, *lpxE* expression strains showed reduced virulence and induced adaptive immunity against antigens in mice (12). *Yersinia pestis* reduces lipid A acylation at 37°C, resulting in a reduction of macrophage activation via tumor necrosis factor alpha (TNF-α) (23). In some bacteria, modification of lipid A charge through the addition of 4-amino-arabinose (Ara4N) or removal of phosphates leads to resistance against cationic antimicrobial peptides like polymyxin B (24, 25). Disruption of lipid A synthesis in *Escherichia coli*, through *lpxA* mutation, reduces the permeability across the outer membrane making the bacteria highly susceptible to antibiotics (26). Gram-negative pathogens have employed numerous lipid A-Kdo modifications, resulting in increased survival and reduced host recognition (20).

*Burkholderia pseudomallei* is a gram-negative bacterial pathogen that causes the disease melioidosis (27). Melioidosis, once thought isolated to Southeast Asia and Australia, is now predicted to be found in 79 countries and cause approximately 89,000 deaths annually (28). Wet tropical soil serves as an environmental reservoir, and exposure occurs through disruptions in cutaneous defenses, inhalation, or ingestion (29, 30). Major rainfall events are often associated with increased cases of melioidosis, likely from exposure to water contaminated with *B. pseudomallei* (29). Human infections often occur in individuals with pre-existing conditions such as diabetes mellitus and can cause acute fulminant infections hallmarked by bacterial sepsis (31–33). Long-term chronic carriage infections have also been reported (34). Overall, melioidosis is a highly adaptable pathogen that can manifest as acute, chronic, or latent infections in the respiratory, gastrointestinal, central nervous, cardiovascular, urinary tract, and musculoskeletal systems (27, 29). While *B. pseudomallei* and other *Burkholderia* spp. encode many virulence factors and have numerous adaptive processes, they are also able to modify the structure of LPS in response to external factors such as temperature or antibiotic exposure. These *Burkholderia* spp. have been shown to produce a penta-acylated lipid A with tetra-acylated sub-populations (35). *Burkholderia pseudomallei* was also shown to cap its lipid A phosphoryl groups with Ara4N. The Ara4N group masks the negative charge of the lipid A phosphoryl groups, eliminating binding sites for the cyclic cationic peptides polymyxin B and colistin, rendering *Burkholderia* spp. resistant to these antimicrobials (36, 37). LPS is unable to be properly assembled at the outer membrane without this group, making it essential for bacterial viability (38). Additionally, sub-populations of *B. pseudomallei* lipid A are singly phosphorylated, reducing the accessible negative charges on the molecule, another mechanism of immunological masking and decreased antimicrobial substrate binding (39, 40). Highly pathogenic *B. pseudomallei* and its closely related non-pathogenic near-neighbor *Burkholderia thailandensis* are physiologically very similar; however, a small but major difference between the two LPS structures was noticed early in the literature: a hydroxyl group addition to the acyl chains in the lipid A of *B. pseudomallei*. This small difference between the two LPS was correlated with a decreased ability of *B. pseudomallei* LPS to activate secretion of inflammatory cytokines through TLR4 activation of host cells compared to *B. thailandensis* LPS (35). LpxO is responsible for the hydroxyl modification of *B. pseudomallei* LPS (41). *Burkholderia thailandensis* does not have an *lpxO* homolog. Preliminary experiments indicated *lpxO* may be upregulated in response to higher temperature (37°C vs 25°C) and contributed to intracellular replication of *B. pseudomallei* via decreased recognition of its LPS (41). Another important lipid A modification gene is *pagL*. PagL was shown to be involved in deacylation of penta-acylated lipid A to tetra-acylated lipid A. Tetra-acylated lipid A has lower binding efficiency to TLR4 (42), lowering recognition of LPS by the mammalian intracellular inflammasome compared to penta- or hexa-acylated lipid A (43). The lipid A of wild-type (WT) *B. pseudomallei* LPS is a variable structure that is mostly tetra-acylated, hydroxylated, and mono-phosphorylated, which reduces TLR4 activation of the host immune system, thus reducing cytokine secretion and associated inflammatory signaling (35, 43).

Outer membrane vesicles (OMVs) are spherical, bilayered, nanosized particles that are naturally produced by gram-negative bacteria under environmental stress and nutrient starvation (44). During active growth, small portions of bacterial outer membrane bleb and pinch off, releasing OMVs. The OMVs contain bacterial outer membrane components such as LPS and capsular polysaccharide (CPS), a minor portion of periplasmic proteins, DNA/RNA, and toxins (45). OMVs can stimulate host innate immune response as they carry several immunogenic substances (e.g., LPS, lipoproteins, and DNA). Due to the non-replicative nature of OMVs, it is an effective carrier for delivering numerous antigens during vaccination. Several studies have demonstrated OMVs as a promising vaccine candidate for pathogenic bacteria such as *Salmonella enterica* Typhimurium (46), *Bordetella pertussis* (47), and *B. pseudomallei* (48, 49). Previously published studies used OMVs from wild-type bacterial strains. To date, there has been no study demonstrating the immunological effects of lipid A modified *B. pseudomallei* OMVs. Therefore, we sought to bioengineer *B. pseudomallei* OMVs by creating a panel of defined lipid A structural modification strains and characterized their impact on innate immune responsivity.

In the present study, a panel of defined mutants and inducible expression strains were created to understand the contribution of *lpxO-*, *pagL-*, and *lpxE-*mediated modifications on membrane permeability in *B. pseudomallei. Burkholderia pseudomallei* does not contain a known LpxE phosphatase; however, a previous study showed that *lpxE* selectively removes the 1-phosphate group from lipid A of *Francisella novicida* (50). An *S. enterica* Typhimurium vaccine with 1-dephosphorylated lipid A exhibited reduced virulence but retained its immunogenic effect against wild-type bacterial challenge (12, 51). This suggests that dephosphorylated lipid A may be a way to enhance vaccine effectiveness against other diseases, such as melioidosis. In this study, the *lpxE* gene of *F. novicida* was codon optimized and expressed in the attenuated *B. pseudomallei* strain Bp82. This would be the first study to investigate the function of *lpxE* in *B. pseudomallei*, which could enable the controlled generation of mono-phosphorylated lipid A (MPLA) in an effort to enhance the development of OMV-based vaccines and/or immune modulators targeting *B. pseudomallei*. We evaluated the immunological effects of the modified OMVs in human macrophages to determine their potential efficacy for incorporation in therapeutics.

## RESULTS

### Modification of lipid A by LpxO, PagL, and LpxE leads to distinct mass profiles in *B. pseudomallei*

**Fig 1:**
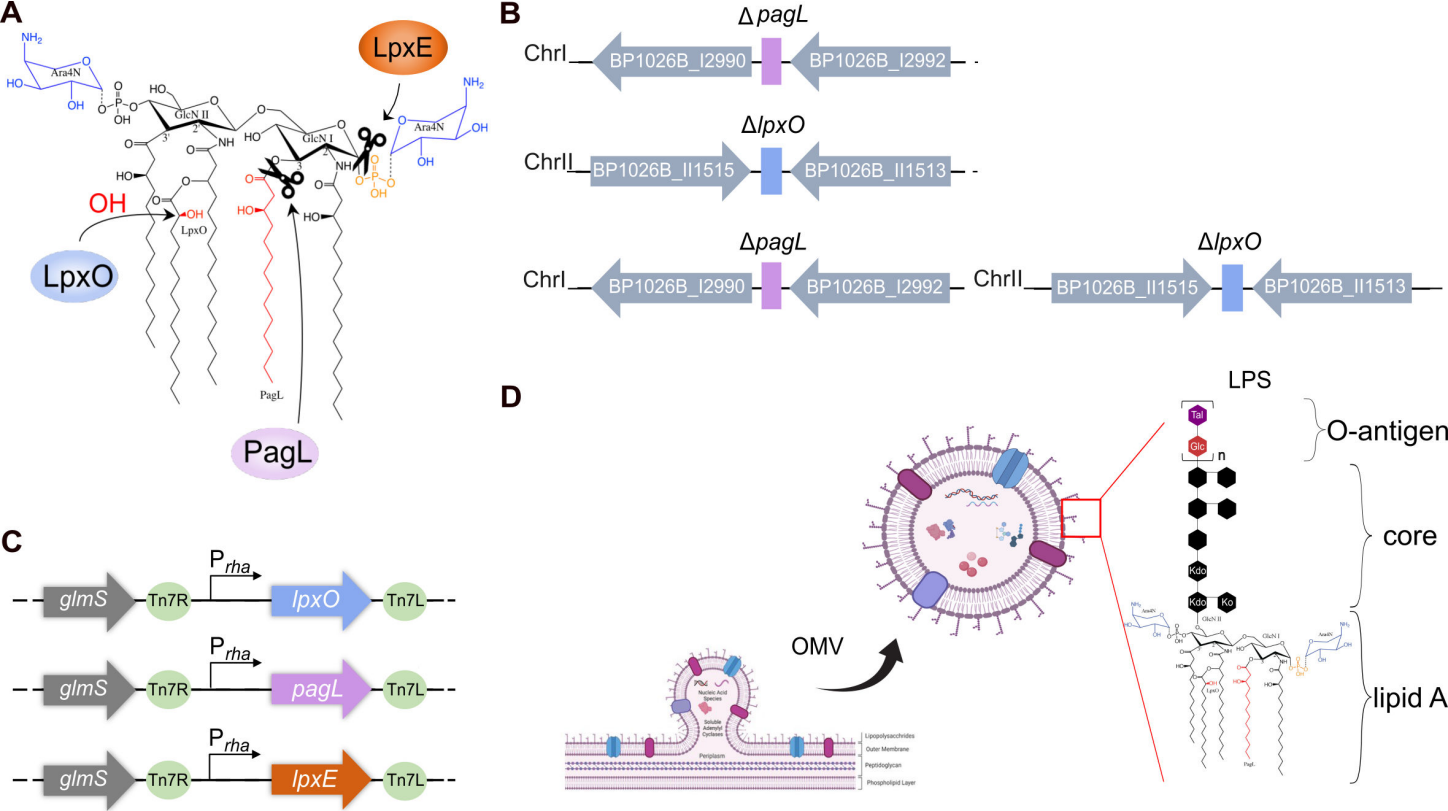
*B. pseudomallei* LPS structure and tunable strains. (**A**) Lipid A structure as determined previously (41). Black arrows show where LpxE, LpxO, and PagL act to modify lipid A by removing a phosphate, adding a hydroxyl, or removing an acyl in red; respectively. (**B**) Markerless knockouts of *lpxO* (Bp82 Δ*lpxO*), *pagL* (Bp82 Δ*pagL*), and the double mutant *lpxO pagL* (Bp82 Δ*lpxO* Δ*pagL*) generated in biosafe *B. pseudomallei* Bp82 strain. (**C**) Tunable complementation strains developed in the present study have rhamnose-inducible promoters driving the expression of *lpxE*, *lpxO*, or *pagL* from *B. pseudomallei* Bp82. (**D**) Outer membrane vesicles produced by gram-negative bacteria contain LPS, CPS, and transmembrane proteins. LPS core structure as described in Ernst et al., 1999 (55).

Previous work has shown that mutation of *lpxO* in *B. pseudomallei* 1026b eliminates hydroxylation of the C_14:0_ esterified to the amide-linked 3-hydroxypalmitate on position 2′ of the distal D-glucosamine (GlcN_II_) residue of the lipid A backbone (Fig. 1A) (41). This mechanism is similar to the function of *lpxO* elucidated in *Salmonella* (52, 53). To better study the physiological role of LpxO in *B. pseudomallei*, an *lpxO* deletion mutant was generated in the attenuated strain Bp82 (Table 1; Fig. 1B). *Burkholderia* spp. produce both tetra- and penta-acylated lipid A (35, 41, 54). It has been shown that the penta-acylated lipid A is the base molecule for *B. pseudomallei* 1026b and that *pagL* is required for deacylation and generation of the tetra-acylated lipid A (Fig. 1A) (41). A *pagL* deletion mutant was generated in *B. pseudomallei* strain Bp82 (Table 1; Fig. 1B). In addition, a double mutant was also generated, removing both *lpxO* and *pagL* (Table 1; Fig. 1B). Bp82 Δ*lpxO*, Bp82 Δ*pagL* single mutants, and the Bp82 Δ*lpxO* Δ*pagL* double mutant were complemented with *lpxO* and/or *pagL* at an *att-*Tn*7* site driven by the rhamnose-inducible promoter (P*_rha_*) (Table 1; Fig. 1C).

**TABLE 1:** Bacterial strains and plasmids

Strain or plasmid	Abbreviation	Reference
Bp82 strains		
Δ*lpxO*	Bp82 Δ*lpxO*	This study
Δ*pagL*	Bp82 Δ*pagL*	This study
Δ*lpxO* Δ*pagL*	Bp82 Δ*lpxO* Δ*pagL*	This study
Bp82 complement strains		
Δ*lpxO glmS2*::P*_rha_-lpxO*	Bp82 *lpxO* comp	This study
Δ*pagL glmS2*::P*_rha_-pagL*	Bp82 *pagL* comp	This study
Δ*lpxO* Δ*pagL glmS3*::P*_rha_-lpxO glmS1*::P*_rha_-pagL*	Bp82 Δ*lpxO* Δ*pagL lpxO* comp *pagL* comp	This study
*lpxE* expressing strains		
Bp82 *glmS1*::P*_rha_-lpxE_Fn-_*_CO_	Bp82 *lpxE*	This study
Bp82 Δ*lpxO* Δ*pagL glmS1*::P*_rha_-lpxE_Fn-_*_CO_	Bp82 Δ*lpxO* Δ*pagL lpxE*	This study
Plasmids		
pEXKm5		(56)
pUC18T-mini-Tn*7*T-Tp-P*_rha_*		(57)
pTNS3		(58)
pFLPe4		(58)

**Fig 2:**
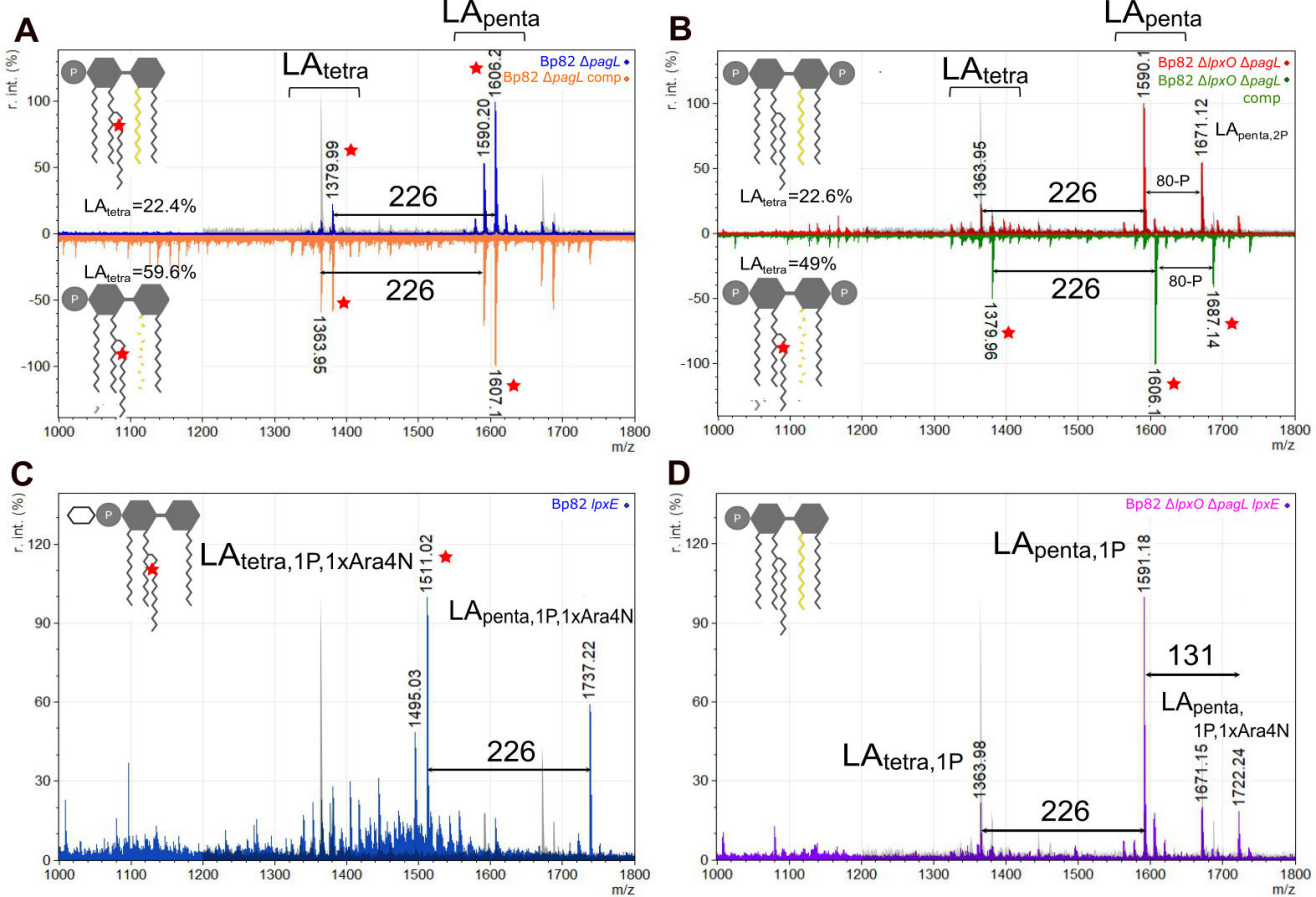
MALDI-TOF of prominent penta-acylated lipid A from *B. pseudomallei* lipid A modification strains. (**A**) Scans from *B. pseudomallei* Bp82 ∆*pagL* (blue) compared to *pagL* complement (Bp82 ∆*pagL* comp) under 0.2% (wt/vol) rhamnose induction (orange). (**B**) Scans from *B. pseudomallei* Bp82 ∆*lpxO* ∆*pagL* (red) compared to *B. pseudomallei* Bp82 ∆*lpxO* ∆*pagL lpxO* comp *pagL* comp under 0.2% (wt/vol) rhamnose induction (green). (**C**) Scans from *B. pseudomallei* Bp82 *lpxE* under 0.2% (wt/vol) rhamnose induction showed a completely mono-phosphorylated lipid A structure (1,511.02 and 1,737.22 *m/z*). (**D**) Scans from *B. pseudomallei* Bp82 ∆*lpxO* ∆*pagL lpxE* under 0.2% (wt/vol) rhamnose induction showed majorly penta-acylated, non-hydroxylated, and mono-phosphorylated lipid A. Gray peaks are scans from WT Bp82 lipid A as described in Norris et al., 2018 (41). Red stars and yellow dashed lines indicate the presence of hydroxyl and absence of acyl chain, respectively.

Mass profiles of lipid A extracts from the deletion mutants and each corresponding complement were analyzed using MALDI-TOF. Deletion of *pagL* from *B. pseudomallei* Bp82 caused a spectrum shift from *m/z* of 1,379.99 (tetra-acylation) to *m/z* of 1,606.20 (penta-acylation) (Fig. 2A, blue). Upon induction of the *pagL* complement strain with 0.2% (wt/vol) rhamnose, a higher proportion of tetra-acylated lipid A was observed with a relative intensity of 59.3%, in comparison to 22.4% of the Bp82 Δ*pagL* strain (Fig. 2A, orange). Deletion of both *lpxO* and *pagL* resulted in a penta-acylated structure lacking a hydroxyl group, which was shown by the spectral peak at 1,590.14 *m/z* (Fig. 2B, red). Complementation and induction of *lpxO* and *pagL* in the double mutant restored the hydroxylation of the tetra-acylated structure to 49% relative intensity compared to 22.6% in the double mutant (Fig. 2B, green). These data show that deletion of *lpxO* and *pagL* alters the mass profiles of lipid A toward more homogenous populations that can help clarify our understanding of *B. pseudomallei* outer membrane physiology.

Our previous study showed that *B. pseudomallei* lipid A is a complex mixture of mono- and di-phosphorylated lipid A (41). There are no known homologs of a lipid A phosphatase in the *B. pseudomallei* genome, and mono-phosphorylated lipid A may be a byproduct observed from laser ionization during MALDI-TOF. Since the homolog is unknown, we could not target this gene for knockout. However, several studies showed that *lpxE* selectively removes a phosphate group at position 1 of GlcN_I_ in lipid A of *Rhizobium leguminosarum* (59)*, F. novicida* (50), and *H. pylori* (60). To better understand how mono-phosphorylation contributes to lipid A properties, the *lpxE* sequence was retrieved from *F. novicida* (WP_003035597.1), codon optimized, and inserted into the genome at the *att-*Tn*7* site in Bp82 and in Bp82 Δ*lpxO* Δ*pagL* (Table 1; Fig. 1C). When *lpxE* is induced from a rhamnose-inducible promoter in Bp82, two major spectral peaks are observed at *m/z* 1,511.02 (mono-phosphorylated hydroxylated tetra-acylated lipid A) and 1,737.22 (mono-phosphorylated non-hydroxylated penta-acylated Ara4N lipid A), indicating successful *lpxE* modification, presumably at position 1 of GlcN_I_ in lipid A pending a more detailed tandem mass spectrometry (MS-MS) analysis of the lipid A structure (Fig. 2C). A similar observation was made when *lpxE* was induced in the Bp82 Δ*lpxO* Δ*pagL* background, with a dominant peak at 1,591.18 *m/z* representing the non-hydroxylated, penta-acylated, and mono-phosphorylated lipid A population (Fig. 2D). There was an additional subpopulation identified in the Bp82 Δ*lpxO* Δ*pagL lpxE* strain of tetra-acyl, non-hydroxyl and mono-phosphorylated lipid A represented by the 1,363.98 *m/z* peak at 21.29% relative to the penta-acylated peak (1,591.18 *m/z*) intensity (Fig. 2D).

### Modification of lipid A by *lpxO* and *pagL* decreases outer membrane permeability in *B. pseudomallei*

**Fig 3:**
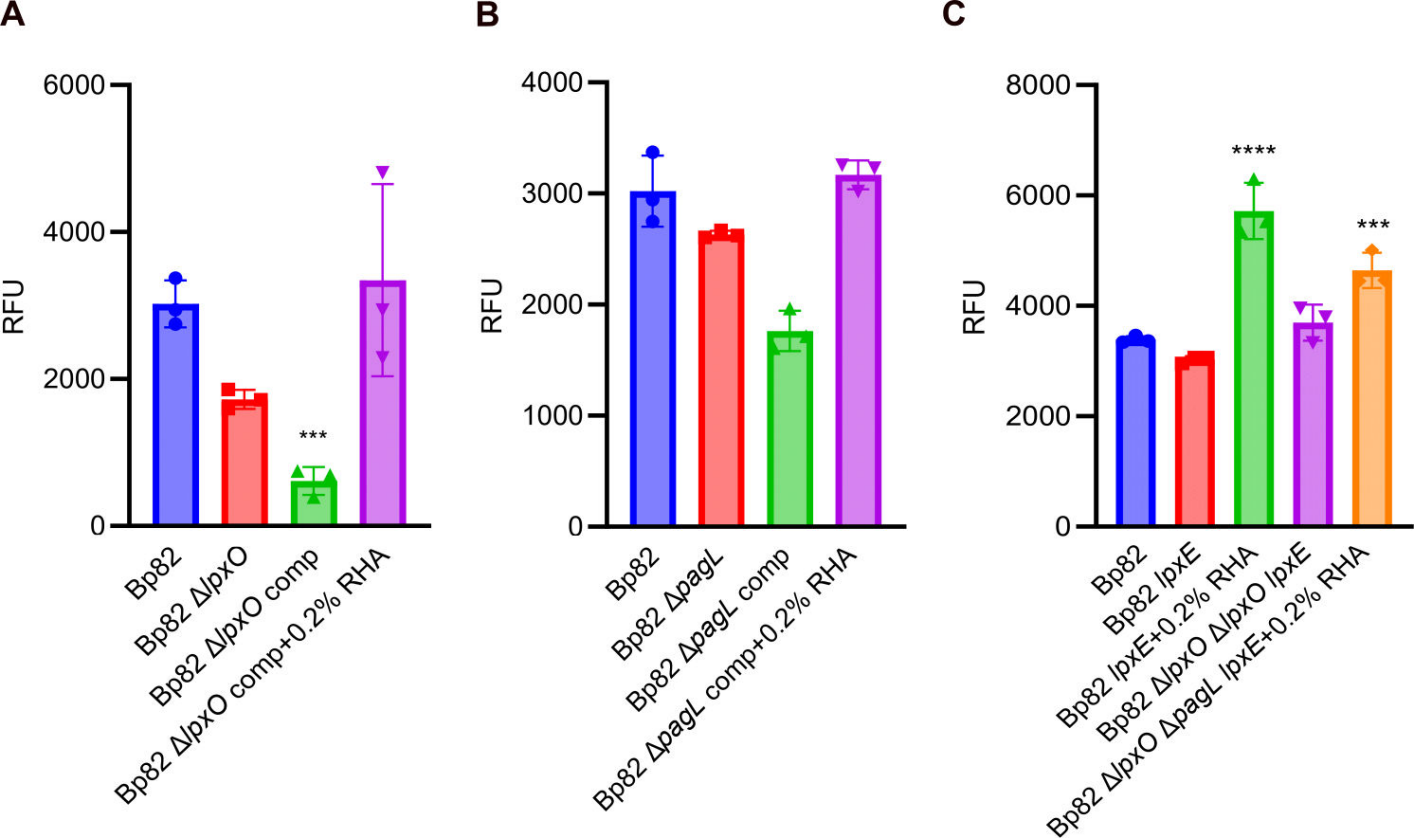
Outer membrane permeability of *B. pseudomallei* strains measured by NPN assay. Complement and *lpxE*-expressing strains were induced with 0.2% (wt/vol) rhamnose (0.2% RHA). (**A**) Fluorescence level was compared between WT Bp82, Bp82 ∆*lpxO*, uninduced *lpxO* complement (Bp82 ∆*lpxO* comp), and induced complement (Bp82 ∆*lpxO* comp + 0.2% RHA) strains. (**B**) Fluorescence level was compared between WT Bp82, Bp82 ∆*pagL*, uninduced *pagL* complement (Bp82 ∆*pagL* comp), and induced complement (Bp82 ∆*pagL* comp + 0.2% RHA) strains. (**C**) Fluorescence level was compared between WT Bp82, uninduced *lpxE*-expressing Bp82 (Bp82 *lpxE*), induced *lpxE*-expressing Bp82 (Bp82 *lpxE* + 0.2% RHA), uninduced *lpxE*-expressing Bp82 ∆*lpxO* ∆*pagL* (Bp82 ∆*lpxO* ∆*pagL lpxE*), and induced *lpxE*-expressing Bp82 ∆*lpxO* ∆*pagL* (Bp82 ∆*lpxO* ∆*pagL lpxE* + 0.2% RHA). The experiment was done in triplicates. Error bar represents standard deviation (SD) of data from three biological replicates. Statistical significance was calculated using Student’s *t*-test, and all strains were compared to WT Bp82 (*P ≤ 0.05, **P ≤ 0.01, *** *P* ≤ 0.001, ****P ≤ 0.0001).

The contributions of lipid A hydroxylation and deacylation on membrane permeability in *B. pseudomallei* are unknown. To study the influence of LpxO and PagL on permeability of the outer membrane, an 1-N-phenylnaphthylamine (NPN) assay was performed on *B. pseudomallei* mutant and complemented strains. The *lpxO* knockout strain showed 9.56% or 11.67% decrease in membrane permeability, compared to the WT *B. pseudomallei* Bp82 or the induced *lpxO* complement strain, respectively (Fig. 3A). Without induction, the *lpxO* complement strain had significantly decreased membrane permeability, compared to WT Bp82, by 17.76% (Fig. 3A). The Bp82 Δ*pagL* strain and the uninduced *pagL* complement strain showed respective 2.86% and 9.27% reductions in membrane permeability when compared to WT Bp82 (Fig. 3B). Induction of *pagL* in the complement strain brought membrane permeability back to WT Bp82 levels (Fig. 3B). However, in both knockout strains, uninduced complement strains showed lower membrane permeability than the uncomplemented mutant strains. Leaky expression of the rhamnose-inducible promoter (P*_rha_*) could lead to low-level expression of the inserted genes. The compensatory low-level lipid A modification may result in a more ordered membrane, allowing tighter membrane lipid packing than the complete absence of expression as found in the deletion mutants or moderate expression levels in the wild type. The result was an observed decrease in membrane permeability compared to the uncomplemented single knockout mutants. These results show a link between membrane permeability and modification of the lipid A backbone by LpxO and PagL.

### Dephosphorylation of lipid A increases membrane permeability

Even though the *B. pseudomallei* genome does not encode obvious lipid A phosphatases, *B. pseudomallei* contains mono-phosphorylated and di-phosphorylated lipid A moieties, obscuring the role of lipid A phosphorylation status in membrane permeability. Addressing this, we measured membrane permeability characteristics of the Bp82 *lpxE* and Bp82 Δ*lpxO* Δ*pagL lpxE* strains using the NPN assay. WT Bp82, uninduced Bp82 *lpxE,* and uninduced Bp82 Δ*lpxO* Δ*pagL lpxE* strains showed slightly different, but not significant, levels of membrane permeability (Fig. 3C). This could be caused by leaky expression of the rhamnose-inducible promoter (P*_rha_*) where *lpxE* was inserted. The leaky expression of *lpxE* in uninduced conditions could impact membrane stability, resulting in variable effects on bacterial membrane permeability, compared to WT Bp82. Upon induction of *lpxE*, there was a significant increase in membrane permeability in the Bp82 *lpxE* strain compared to WT Bp82 (Fig. 3C). Expression of *lpxE* in the Bp82 Δ*lpxO* Δ*pagL* background also significantly increased membrane permeability but to a lower level than observed in the WT Bp82 *lpxE* expression strain (Fig. 3C). This is likely due to the additive effects of deleting *lpxO* and *pagL*, which decreases membrane permeability as observed in Fig. 3A and B. These results indicate that dephosphorylation by LpxE increases membrane permeability of *B. pseudomallei*, and this effect can be partially quenched by mutation of *lpxO* and *pagL*.

### *lpxE*-mediated modification of *B. pseudomallei* OMV increases immunomodulatory expression in macrophages

**Fig 4:**
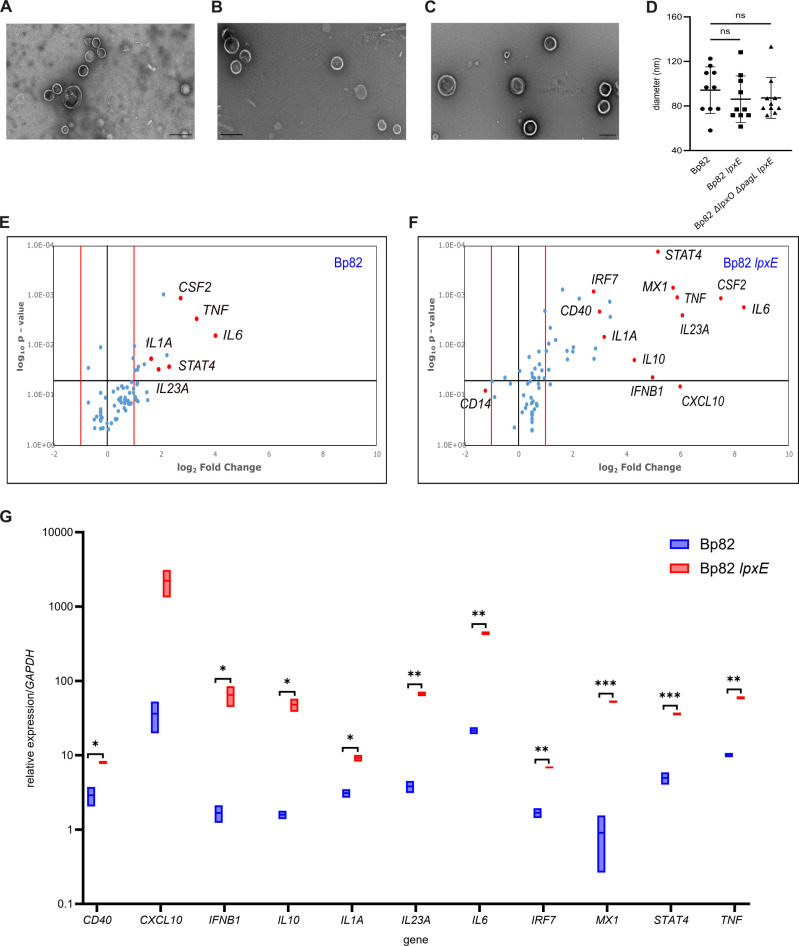
Gene expression profile upon OMV treatment in human THP-1-derived macrophages. Transmission electron microscope (TEM) images of purified OMVs from WT Bp82 (**A**), Bp82 *lpxE* (**B**), and Bp82 Δ*lpxO* Δ*pagL lpxE* (**C**). The micrograph shows OMVs 50–200 nm in diameter with no morphological differences between WT and mutant strains (scale bar = 200 nm). (**D**) Diameter of 10 OMVs from each strain was measured in Fiji, and there was no significant difference in size of OMVs between the WT and mutants. Log_2_FC gene expression upon treatment of THP-1 derived macrophages with (**E**) Bp82 OMV and (**F**) Bp82 *lpxE* OMV, compared to the untreated control group. Differential expression level cutoff was defined as log_2_FC ≥ 1. Red dots indicate differentially expressed genes. (**G**) Gene expression compared between WT Bp82 and Bp82 *lpxE* OMV-treated groups at 4 h after treatment. *GAPDH* was used as a housekeeping gene for the analysis. Expression level was calculated using RT^2^ profiler PCR array template sheet for human innate and adaptive immune responses as per instructions. Two independent experiments were done in triplicate. Statistical significance was calculated using Student’s *t*-test (*P ≤ 0.05, **P ≤ 0.01, *** *P* ≤ 0.001, ****P ≤ 0.0001).

OMVs are a promising candidate for a melioidosis vaccine because they contain numerous antigens capable of stimulating an immune response and are non-replicating (Fig. 1D) (48, 49). MPLA has been shown to be an effective vaccine adjuvant due to low endotoxicity and high immunogenicity (51, 61–63). Our results showed that Bp82 *lpxE* LPS and/or the Bp82 Δ*lpxO ΔpagL lpxE* LPS increased gene expression of inflammatory cytokines tumor necrosis factor (*TNF*) and interferon-gamma (*IFNG*) in human macrophages, compared to WT Bp82 LPS (Fig. S1). To test the immunomodulatory role of OMVs with a mono-phosphorylated lipid A, OMVs were isolated from WT Bp82, Bp82 *lpxE*, and Bp82 Δ*lpxO ΔpagL lpxE*. OMVs were visualized using transmission electron microscopy (TEM), showing multiple bilayered particles 50–200 nm in diameter, consistent with previously reported sizes of OMVs (64) (Fig. 4A through C). OMVs extracted from WT Bp82 and mutant strains showed no significant differences in morphology (Fig. 4A through C) and size (Fig. 4D). This indicates that expression of *lpxE* causes no morphological changes in *B. pseudomallei* OMVs. Subsequently, OMVs derived from each strain background were used to treat THP-1-derived macrophages. Gene expression was measured four hours post-treatment by real-time PCR using Human Innate & Adaptive Immune Responses RT² Profiler PCR Arrays (Table S2). We found that upon treatment with Bp82 OMVs, interleukin-6 (*IL6*), tumor necrosis factor (*TNF*), colony-stimulating factor 2 (granulocyte-macrophage) (*CSF2*), signal transducer and activator of transcription 4 (*STAT4*), interleukin 23-alpha subunit p19 (*IL23A*), and interleukin-1α (*IL1A*) were significantly upregulated (Fig. 4E). In the group treated with Bp82 *lpxE* OMV, *IL6*, *CSF2*, *IL23A*, CXC motif chemokine ligand 10, *TNF*, interferon-inducible protein p78 (*MX1*), *STAT4*, interleukin-10 (*IL10*), interferon beta (*IFNB1*), *IL1A*, interferon regulatory factor 7 (*IRF7*), and TNF receptor superfamily member 5 (*CD40*) were significantly upregulated, compared to untreated controls (Fig. 4F). Following comparison between Bp82 OMV-treated and Bp82 *lpxE* OMV-treated THP-1 cells, gene expression of *CD40, IFNB1*, *IL10*, *IL1A*, *IL23A*, *IL6*, *IRF7*, *MX1*, *STAT4*, and *TNF* were significantly higher in the Bp82 *lpxE* OMV-treated THP-1 cells (Fig. 4G).

**Fig 5:**
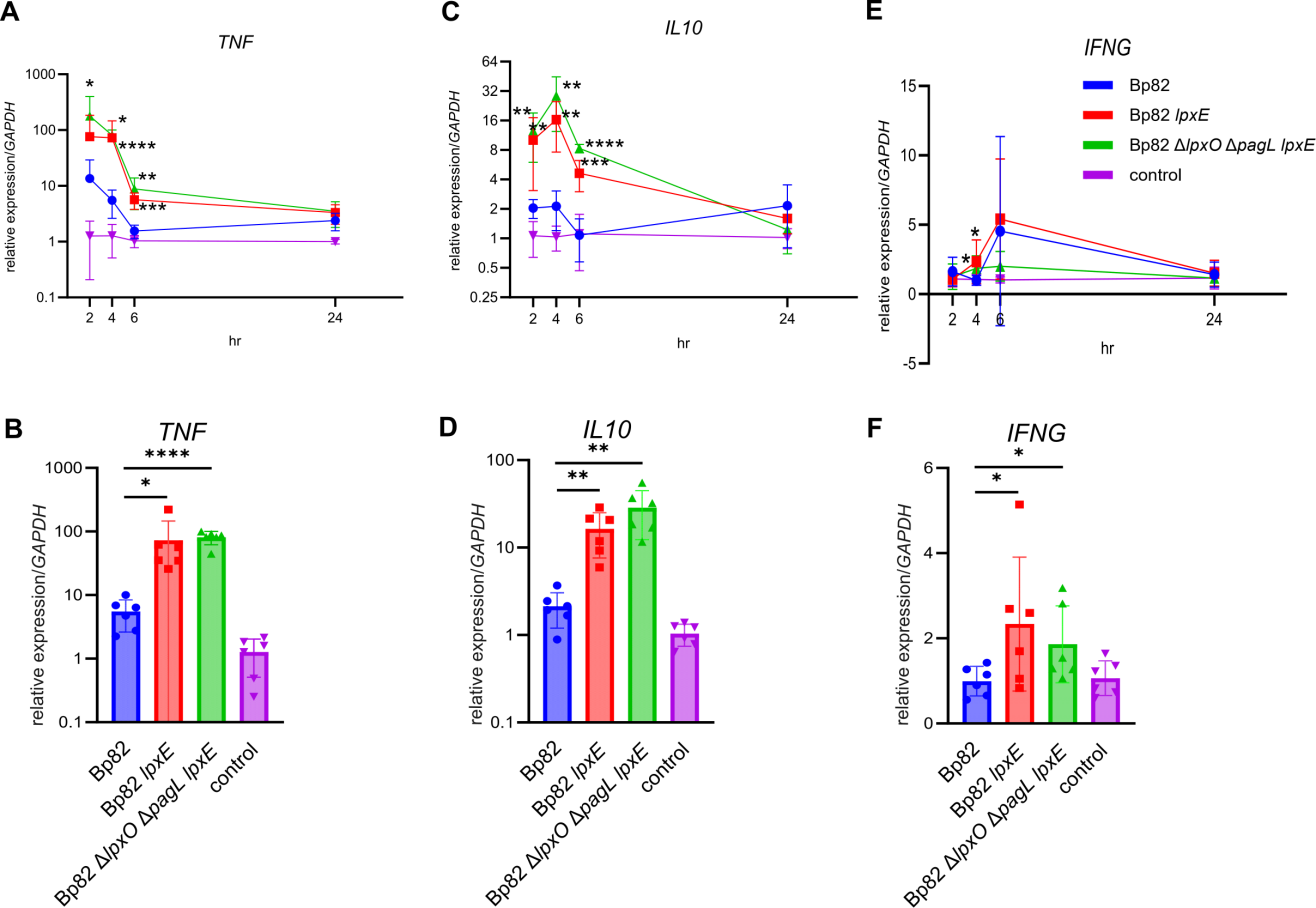
Validation of cytokine gene expression upon treatment of macrophages with OMV from *lpxE*-expressing *B. pseudomallei*. Expression of *TNF* (**A and B**), *IL10* (**C and D**), and *IFNG* (**E and F**) in THP-1-derived macrophages after treatment with OMV extracted from WT Bp82, Bp82 *lpxE,* and Bp82 Δ*lpxO* Δ*pagL lpxE* strains as quantified by real-time PCR. (**B, D, and F**) Expression of *TNF*, *IL10,* and *IFNG*, respectively, at 4 h post-treatment. *GAPDH* was used as an internal control. Relative expression was calculated using the Pfaffl method. Error bars represent standard deviation (SD) of data from two independent experiments with three biological replicates each. Statistical significance was calculated by comparison to WT Bp82 using Student’s *t*-test, (*P ≤ 0.05, **P ≤ 0.01, *** *P* ≤ 0.001, ****P ≤ 0.0001).

To analyze how gene expression changes over time, the gene expression of three important immunomodulatory cytokines, *TNF*, *IL10*, and *IFNG*, was measured for 24 h in THP-1-derived macrophages following treatment with OMVs. Bp82 *lpxE* and Bp82 Δ*lpxO* Δ*pagL lpxE* OMVs induced significantly higher expression of *TNF* in THP-1-derived macrophages during the first 6 h of the 24-h time course in comparison to WT Bp82 OMV and the untreated control (Fig. 5A and B). In all treatment groups, the expression of *TNF* was highest at 2 h and then gradually decreased at the 24 h timepoint (Fig. 5A). Over the course of the experiment, *TNF* expression remained higher in groups treated with Bp82 *lpxE* and Bp82 Δ*lpxO* Δ*pagL lpxE* OMVs than in those of Bp82 OMVs and the untreated control groups (Fig. 5A). At 4 and 6 h post-treatment, THP-1-derived macrophages treated with OMVs derived from an *lpxE* expression background showed significantly higher expression of *TNF* when compared to macrophages treated with WT Bp82 OMVs or the untreated control (Fig. 5A). These results indicate that both pure LPS and OMV from Bp82 Δ*lpxO* Δ*pagL lpxE* similarly upregulated expression of *TNF* in human THP-1-derived macrophages, compared to that of WT Bp82 LPS and OMV, and the untreated control group (Fig. S2A).

Modulation of *IL10* expression was also observed in THP-1-derived macrophages treated with various OMVs. Expression of *IL10* was significantly elevated at early time points when macrophages were treated with OMVs derived from Bp82 *lpxE* and Bp82 Δ*lpxO* Δ*pagL lpxE* when compared to WT Bp82 (Fig. 5C). Unlike *TNF* expression, *IL10* expression peaked at 4 h post-treatment in all groups treated with an OMV (Fig. 5C). At 4 h post-treatment, *IL10* expression in the groups treated with Bp82 *lpxE* and Bp82 Δ*lpxO* Δ*pagL lpxE* OMV was significantly higher than the Bp82 OMV and untreated control groups (Fig. 5D). At 6 h post-treatment, *IL10* expression subsided in all OMV-treated groups; however, those treated with Bp82 *lpxE* and Bp82 Δ*lpxO* Δ*pagL lpxE* OMV had significantly elevated expression compared to the Bp82 OMV-treated group (Fig. 5C). While there was no difference in expression of *IL10* between LPS derived from WT Bp82- and *lpxE*-expressing strains, the significant upregulation of *IL10* upon treatment with Bp82 *lpxE* and Bp82 Δ*lpxO* Δ*pagL lpxE* OMV suggests that other components of OMV, such as proteins, might drive this different response (Fig. S2B).

*IFNG* also showed significant changes in expression after THP-1-derived macrophages were treated with various OMVs. However, when compared to *TNF* and *IL10*, fewer significant changes to *IFNG* expression were observed in macrophages (Fig. 5). There were no significant changes in *IFNG* expression observed for any treatment group at 2, 6, or 24 h post-treatment (Fig. 5E). At 4 h post-treatment, THP-1-derived macrophages treated with Bp82 *lpxE* and Bp82 Δ*lpxO* Δ*pagL lpxE* OMVs showed a significant increase in *IFNG* expression (Fig. 5E and F). There was no significant change observed in *IFNG* expression at 4 h when comparing macrophages treated with WT Bp82 OMVs and the untreated control (Fig. 5F). At 6 h post-treatment, there was an increase in *IFNG* expression in macrophages treated with WT Bp82 and Bp82 *lpxE* OMVs; however, this change was not statistically significant (Fig. 5E). From both LPS and OMV treatment in human THP-1-derived macrophages, our findings showed that pure LPS and OMV from Bp82 *lpxE* strain significantly induced expression of *IFNG*, compared to WT Bp82 LPS and OMV-treated and control groups (Fig. S2C). Together, OMVs derived from an *lpxE* expression background, including Bp82 *lpxE* and Bp82 Δ*lpxO* Δ*pagL lpxE*, significantly modulate the expression of *TNF*, *IL10*, and *IFNG*, suggesting that these OMVs could be used as an immune modulator in vaccines against melioidosis.

## DISCUSSION

*Burkholderia pseudomallei* is a complex organism that is able to survive in diverse environments, including soils, water, and within living organisms. Its large genome confers many mechanisms that allow it to adapt and thrive in these environments, including modification of lipid A. While lipid A modifications have been studied extensively in model organisms like *Escherichia coli* and *Salmonella*, there is less understanding of how changes to the outer membrane affect organisms like *B. pseudomallei*. Previous work has shown that LPS from *B. pseudomallei* is less immunogenic than LPS from *B. thailandensis*, a closely related but less-pathogenic species (35). This difference is partially due to LpxO-mediated hydroxylation of the C_14:0_ acyl chain because *B. thailandensis* lacks an *lpxO* homolog (35, 41). PagL modifications are also known to reduce TLR4 activation by reducing NFκB expression, suggesting that a shift in lipid A subpopulations from penta-acylation to tetra-acylation helps avoid host defenses (65). Here, we confirm the structural modifications made by LpxO and PagL through mutation and complementation analysis and give insights into their role in *B. pseudomallei* membrane permeability.

In the present study, modifications to the lipid A backbone by deletion of *lpxO* or *pagL* were found to influence physiological characteristics of the *B. pseudomallei* outer membrane. The results showed that *B. pseudomallei lpxO* and *pagL* mutants produce non-hydroxylated and penta-acylated lipid A, moving the lipid A subpopulations in these strains toward more homogeneous mixtures. The deletion of *lpxO* or *pagL* had a similar effect on the membrane permeability of *B. pseudomallei*. Lack of hydroxylation through LpxO reduced the permeability of the *B. pseudomallei* outer membrane. Similarly, shifting the lipid A subpopulations toward penta-acylation by deletion of *pagL* also reduces *B. pseudomallei* membrane permeability. These modifications can play major roles in the physiological function of the outer membrane. Decreased membrane permeability could be achieved through regulatory control of *lpxO* and/or *pagL* in *B. pseudomallei* that could lead to exclusion of antibiotics or other toxic substances from entering the cell (20).

LpxE is a phosphatase first identified in *Rhizobium leguminosarum* and is responsible for lipid A 1-dephosphorylation (59). Dephosphorylation of lipid A to MPLA is known to reduce endotoxicity while maintaining immunogenicity (66). While *B. pseudomallei* does not encode any known lipid A phosphatases, it has been shown to have both di-phosphorylated and mono-phosphorylated lipid A subpopulations (41). In this work, we sought to investigate how mono-phosphorylation contributed to membrane permeability of *B. pseudomallei*. Structurally, we found that induction of *lpxE* removed one phosphate group from lipid A, shifting the lipid A to a completely mono-phosphorylated structure. This indicates a conserved function of the *lpxE* gene across various bacterial species. These modifications also caused changes to *B. pseudomallei* outer membrane permeability. While dehydroxylation and acylation reduced membrane permeability of *B. pseudomallei*, dephosphorylation increased it. Importantly, the variations in membrane permeability and the modification of outer membrane charge could alter TLR4/MD-2 signaling during host infection.

There is currently no licensed vaccine for melioidosis, although much effort has been expended towards this goal (67). OMVs have been an avenue of increased interest because they are immunostimulatory, non-replicating and contain numerous antigens (68). OMVs derived from WT *B. pseudomallei* 1026b induced T cell memory responses and provided antibody-mediated protection against acute and lethal melioidosis infection (48, 69). OMVs contains the outer leaflet of the outer membrane where lipid A moieties are located. Lipid A modifiers and the diverse subpopulations of *B. pseudomallei* lipid A open up a unique opportunity for fine-tuned immune modulation using lipid A engineering. Mutation of *lpxO* and *pagL* modify the lipid A backbone to a more homogeneous structure, as demonstrated in this work. An *lpxO* and *pagL* double mutant further shifts lipid A profiles to a more consistent population. Expression of *lpxE* in the *lpxO* and *pagL* double mutant background leads to a highly homogeneous lipid A population with a single phosphate subgroup, comparable to MPLA. MPLA has proved to be a safe and effective vaccine adjuvant for decades due to its low toxicity and immunostimulatory properties (62). MPLA triggers production of various cytokines via modification of TLR4 recognition, altering TNF, IL10, and IFN-γ levels (61, 63). We demonstrated how lipid A modifications can modulate innate immune responses and proffer the use of these OMVs to enchance vaccine and adjuvant development in preventing melioidosis.

Mutants were created in this study using the biosafe strain of *B. pseudomallei* 1026b, which exhibits type A LPS because OMVs from this strain have been well-characterized (48, 49, 69). However, expression of *lpxE* and knockout of *lpxO* and *pagL* are expected to have similar effects on the LPS type B given the similarity in lipid A structure and known functions of *lpxO* and *pagL* in the *B. pseudomallei* 576a type B LPS strain (41, 54). We found that OMVs isolated from *lpxE*-expressing *B. pseudomallei* induced THP-1-derived macrophages to upregulate several immunomodulatory genes compared to WT Bp82 OMV-treated and untreated control groups. Bp82 *lpxE-*expressing OMVs promptly and significantly triggered immune responses, compared to WT Bp82 OMVs, by upregulating proinflammatory cytokines such as *TNF*, *IL6,* and *IL1A*. Additionally, the *lpxE*-modified OMV exhibited enhanced expression of *IFNG* and *IFNB1*. IFN-γ is characteristic of producing strong cellular T_H_1 immunity that induces antibody class switching and affinity maturation to IgG2a (70). IFN-γ production is known to be a key factor of cross-protection in the BCG vaccine (71) and has a correlative link to survival in melioidosis patients (72). IFN-β is a type I interferon (type I IFN). A previous study showed an important role for type I IFN in promoting production of memory-like B cells and specific antibodies in a viral vector vaccine (73). These results indicated that the mono-phosphorylation phenotype of OMVs generated from *lpxE*-expressing strains enhances immunostimulation of macrophages.

OMVs derived from Bp82 *lpxE* and Bp82 Δ*lpxO* Δ*pagL lpxE* strains increased expression of proinflammatory cytokines during a time course experiment, indicating that they could be beneficial in vaccines or adjuvants. Higher expression of cytokines was observed in the Bp82 Δ*lpxO* Δ*pagL lpxE* OMV-treated group. This is likely due to the increased number of acyl chains in the *pagL* knockout strain, which enhances host recognition through activation of TLR4 signal transduction, as observed in other studies (74, 75). OMVs generated from an *lpxE*-expression strain generated immunostimulatory signals, suggestive of a more balanced immune response compared to WT Bp82 OMVs if it were to be used as a vaccine or vaccine adjuvant. During infection, excessive pro-inflammatory cytokines produced by various CD4^+^ T cell subsets can cause pathologies (76, 77). When macrophages were treated with OMVs derived from both *lpxE*-expression backgrounds, there was significantly increased expression of *TNF* at early time points and increased expression of *IFNG* at 4 h post-treatment. The expression of these proinflammatory cytokines was coupled with significant increases in *IL10* expression, peaking at 4 h post-treatment. IL10 plays an essential role in balancing immune responses and is a known anti-inflammatory cytokine acting through suppression of a variety of inflammatory cytokines (78, 79). The engineered OMVs showed significantly increased expression of *IL10*, *TNF*, and *IFNG* when compared to that of the WT Bp82 OMV, suggesting that a strong but balanced immune response was induced.

In this study, we developed strains that generate homogeneous mixtures of lipid A with a single phosphate. We determined that functional LpxO and PagL both increase membrane permeability, suggesting that modulation of *lpxO* or *pagL* expression could help *B. pseudomallei* adapt to different environments. The function of LpxE was confirmed to remove a phosphate in the *B. pseudomallei* lipid A backbone and led to an increase in membrane permeability. In conclusion, OMVs with modified lipid A backbones showed increased immunostimulatory effects on macrophages, suggesting the engineered OMVs may enhance immune activation and warrant future *in vivo* vaccination and challenge testing.

## MATERIALS AND METHODS

### Bacterial strains, cell lines, and growth conditions

Luria-Bertani (LB) containing 0.6 mM adenine was used to cultivate *B. pseudomallei* Bp82 strains at 37°C (80). THP-1-derived macrophages (600,000 cells/mL) were differentiated with 100 ng/mL phorbol 12-myristate-13-acetate (PMA) at 37°C with 5% CO_2_ until fully differentiated (approximately 72 h [81]). Differentiated cells were washed with 1× phosphate-buffered saline (PBS), and the differentiation medium was replaced by RPMI containing 10% fetal bovine serum and 1× antibiotic-antimycotic (without PMA) a day prior to subsequent experiment.

### Construction of *lpxO* and *pagL* knockout and complement strains

*lpxO* and *pagL* from *B. pseudomallei* Bp82 were knocked out using markerless allelic replacement as previously described (56, 58). Briefly, approximately 1,000 bp upstream and downstream fragments of *lpxO* and *pagL* were amplified from Bp82 using *lpxO*-UP and *lpxO*-DWN and *pagL*-UP and *pagL*-DWN primers, respectively, and assembled into pEXKm5 plasmid using Gibson Assembly Master Mix (New England Biolabs). The plasmid was transferred into Bp82 by conjugation. Mutant clones were selected using *sacB*-mediated counterselection and confirmed by Sanger sequencing. For complement strains, coding sequences of *lpxO* and *pagL* from Bp82 were amplified using *lpxO*-comp and *pagL*-comp primers, respectively, and were cloned into pUC18T-mini-Tn*7-FRT*-Tp-P*_rha_* plasmid under a P*_rha_* rhamnose-inducible promoter, and inserted into the mutant strains (58).

To generate *lpxE*-expressing strains, the lipid A 1-phosphatase (*lpxE*) coding sequence retrieved from *F. novicida* (WP_003035597.1) was codon optimized for *B. pseudomallei* codon usage and synthesized by Integrated DNA Technologies (IDT) (Table S1). The synthesized sequence was amplified using *lpxE* primers, cloned into the mini-Tn*7* single-copy complementation system, and inserted into WT Bp82 and Bp82 Δ*lpxO* Δ*pagL* strains using the same strategy described above for *lpxO* and *pagL*.

### LPS isolation and MALDI-TOF MS analysis of lipid A

Isolation of LPS and lipid A preparation were carried out using the hot phenol extraction method (54) with modifications. Briefly, bacterial strains were spread on agar plates and incubated for 3–4 days. The complement and *lpxE* expression strains were grown with addition of 0.2% (wt/vol) rhamnose. Bacterial lawns were suspended in Milli-Q water and heat-inactivated at 100°C for 30 min. An equal volume of 90% phenol was added and slightly stirred at 68°C for 30 min. The mixture was then dialyzed in a tubing with 7 kDa cutoff against water for 4–5 days. After dialysis, the sample was treated with DNase I and RNase A for 2 h and then proteinase K for 2 h at 37°C. After lyophilization, the dried sample (crude LPS) was resuspended in 1 M acetic acid and heated at 110°C for 1.5 h to hydrolyze lipid A. The hydrolyzed sample was lyophilized and purified using the Bligh-Dyer method (82). Chloroform phase containing lipid A was collected and lyophilized. Analysis of lipid A samples was carried out via the MALDI-TOF MS service at the Complex Carbohydrate Research Center.

### Membrane permeability NPN assays

Cell permeability was performed in a Biotek Synergy HTX microplate reader in fluorescence mode as previously described with modifications (83). Bacterial strains were inoculated until optical density at 600 nm (OD_600_) reached 0.5. The complement and induction strains of *lpxE*, *lpxO*, and *pagL* were induced with 0.2% (wt/vol) rhamnose. Bacteria were washed and resuspended in HEPES buffer (5 mM HEPES, pH 7.0) and then adjusted to OD_600_ of 0.5. NPN was added to a final concentration of 10 µM. Fluorescence signal was measured with excitation of 360/40 nm and emission of 460/40 nm.

### Outer membrane vesicle (OMV) extraction

OMV was extracted as previously described (84). Briefly, an overnight culture of bacterial strains was diluted 1:100 into 400 mL fresh LB containing 0.6 mM adenine (complement strains were induced with 0.2% (wt/vol) rhamnose) and cultured for 3–4 days at 37°C. Bacterial cells were harvested by centrifugation at 4,000 × *g* for 15 min. The culture supernatant was filtered through 0.45 and 0.22 µm membranes, respectively. The filtrate was centrifuged at 38,000 × *g* for 2 h at 4°C. Pellet was resuspended in 1 mL PBS. The sample was subsequently overlaid on OptiPrep 45%–20% and separated by centrifugation at 280,000 × *g* for 3 h at 4°C. Each layer was collected, diluted with PBS, and centrifuged at 280,000 × *g* for 2 h at 4°C. The pellet was resuspended in 100 μL PBS and stored at −80°C. Total protein content was quantified using Bradford’s assay (Bio-Rad).

### Transmission electron microscopy (TEM) of OMV

Extracted OMV samples were diluted and placed on a coated copper grid and negatively stained with 2% uranyl acetate. Images were captured using a 120 kV Hitachi HT7700 Transmission Electron Microscope at the Biological Electron Microscope Facility, University of Hawai’i at Manoa. OMV diameter was measured in Fiji; 10 OMVs were randomly picked from 2 images of each strain (5 OMVs/image).

### Total RNA extraction and gene expression analysis using real-time PCR

To determine cytokine expression under OMV treatment, THP-1-derived macrophages were treated with 0.01 μg/mL OMV for 2, 4, 6, and 24 h. Total RNA was extracted using TRIzol reagent (Invitrogen) following manufacturer’s instructions. The extracted RNA was qualified and quantified using a spectrophotometer and agarose gel electrophoresis, respectively. Gene expression at 4 h post-treatment was analyzed using RT^2^ Profiler PCR Array Human Innate & Adaptive Immune Responses (Qiagen) following manufacturer’s instructions. Briefly, the extracted RNA samples were cleaned up using RNase-free DNase set (Qiagen) and converted into cDNA using RT^2^ First Strand Kit (Qiagen). The real-time PCR assay was performed on CFX Connect Real-Time PCR System (Bio-Rad). The RT2 Profiler PCR Array results were analyzed following the manufacturer’s recommendations. For validation of cytokine expression, 50 ng of total RNA was used for quantitative PCR (qPCR) of specific immune gene targets using Verso 1-step RT-qPCR Mix (Thermo Scientific). Glyceraldehyde 3-phosphate dehydrogenase (*GAPDH*) was used as an internal control. Relative expression level was calculated using the Pfaffl method (85). Two technical replicates were conducted with biological triplicates. Statistical significance was calculated by comparison to WT Bp82 using Student’s *t*-test. All primers used are listed in Table 2.

**TABLE 2:** Primers used in this study

Primer	Forward (5′−3′)	Reverse (5′−3′)
*lpxO*-DWN	TGTTATCCCTACCCGGGCGGCCGCCT TTGCGAC TATCGTTATAAGAACCCGG	GGGGGAAAAAATGTGAGCGATCGGTCGCGG
*lpxO*-UP	ACCGATCGCTCACATTTTTTCCCCC GTTACAGCG	CTCTAGGGATAACAGGGTAATCCCGATGTTG ACGACATCTTTGTTGATGAGGAG
*pagL*-DWN	TGTTATCCCTACCCGGGCGGCCGC CATGCGATT TGCGCGC	GGAGTCAGAGATGTGATCGGCGCGCCGGAC
*pagL*-UP	GCGCGCCGATCACATCTCTGACTC CAACTTCTC TGGTTGAATAGAGC	CTCTAGGGATAACAGGGTAATCCCGATAAAAA AGCCCGCCGTCC
*lpxE*	TGAAATTCAGCCAGCAGGATCACA CCATGGCCA TGGGCCTCAAGCAGAC	CGCGAGGTACCGGGCCCAAGCTTCTCGAGG AATTCCTAGATGATCTCCCTGTTCCTCA
*pagL*-comp	GTCAGCCATGGACGATAAGAACGGCG	GCGGAATTCAGAAGTTGTATTGCAC
*lpxO*-comp	GGGAAACCATGGGTTGGGCCATT CTGTTC	GCGAGAATTCGCTCAGCGCCACAGAATC
*TNF*	CATCTATCTGGGAGGGGT	GCGTTTGGGAAGGTTGGAT
*IFNG*	TCGGTAACTGACTTGAATGTCCA	TCGCTTCCCTGTTTTAGCTGC
*IL10*	GACTTTAAGGGTTACCTGGGTTG	TCACATGCGCCTTGATGTCTG
*GAPDH*	GGAGCGAGATCCCTCCAAAAT	GGCTGTTGTCATACTTCTCATGG

For LPS treatment, 1 μg/mL LPS was used to treat THP-1-derived macrophages. After 4 h post treatment, total RNA was extracted, and gene expression was quantified as described above. The experiment was conducted in biological triplicate.

### Statistical analysis

All experiments were conducted in triplicates, and data were reported as mean ± standard deviation. Statistical significance was analyzed by Student’s *t*-test. Statistics were done using GraphPad Prism. Statistical significance was defined at *P* < 0.05.

## SUPPLEMENTAL MATERIAL

Supplemental material iai.00602-25-s0001.docx Tables S1 and S2; Fig. S1 and S2.

ASM does not own the copyrights to Supplemental Material that may be linked to, or accessed through, an article. The authors have granted ASM a non-exclusive, world-wide license to publish the Supplemental Material files. Please contact the corresponding author directly for reuse.
