# Supplementary material for: Outer membrane engineering through lipid A modification allows modulation of Burkholderia pseudomallei membrane permeability and innate immune responsivity
Source: Infect Immun. 2026 Jul 22;94(8):e00602-25. doi: 10.1128/iai.00602-25 (PMC13460123; doi:10.1128/iai.00602-25)
Supplement: Supplementary file 1 [file iai.00602-25-s0001.docx]

**Supplemental material**

**Table S1. Original sequence of *lpxE* from *Francisella novicida* and its codon-optimized sequence for insertion into *B. pseudomallei***

| *F. novicida* *lpxE* | Codon-optimized *lpxE* |
| --- | --- |
| ATGCTCAAACAGACATTACAAATAAACTTTCAAGGTTTTAAAGATATTTTTAAAAAACCAAAACTACACAATCATAAATTGCCTAGATATCTACAGTTGAAATATACGTTTATACCATTATTAATTTTGGTAATTTTTGCATACTATAACTTAGATACCCCAGTTGAGAACTATATCAAGCATTCTATGCCAAATATTGTTGGTGTAATTTTTGGTAAAATAACTGATGTTGGTAAGGCCGAGTATATTTTGATAATTTGCGGTGTGATAGTGTTAGCGCGTTTATTTACAGATAGCCAAAAATTATCTGCTAATGCTAGAGCTATGTTTGACAAGGTGTCGGCATATGCCGGTTTTATCTTAGCAACTGTAGCTATTAGTGGTATTTTGGGACAAATACTCAAGATGATAATAGGTAGAGCGCGTCCTAAGTTTTTCTTGGAATATGGTTCGCATTATTTCCAACATTTTCATGCACCTGGATATGATTTTGCAAGTATGCCGTCAGGGCACTCAATCACAGTTGGAGCAATGTTTATAGCATTTTTTTATATTTTCCCTAAGCTAAGATATTTTTGGTATTTGCTGATAGTGGTATTTGCTGGGAGTAGAATTATAGTTGGTTCACATTATCCTAGTGATGTAATTTTTGGCGTTGCTTTTGGTTGTTACTGTACAGCATATATCTACTATTGGATGAGAAATAGAGAGATTATTTAG | ATGGGCCTCAAGCAGACGCTGCAGATAAACTTCCAGGGTTTCAAGGACATCTTCAAGAAGCCGAAGCTGCACAACCATAAGCTGCCGCGCTACCTGCAGCTGAAGTACACGTTCATCCCGCTGCTGATTCTGGTGATCTTCGCGTACTACAACCTGGACACGCCGGTGGAGAACTACATCAAGCATAGCATGCCGAACATCGTGGGCGTGATCTTCGGCAAGATCACGGACGTGGGCAAGGCGGAGTACATCCTGATCATCTGCGGCGTGATCGTGCTGGCGCGCCTGTTCACGGACAGCCAGAAGCTGAGCGCGAACGCGAGGGCGATGTTCGACAAGGTGAGCGCGTACGCGGGCTTCATCCTGGCGACGGTGGCGATCAGCGGCATCCTGGGCCAGATCCTGAAGATGATCATCGGCAGGGCGAGGCCGAAGTTCTTCCTGGAGTACGGCAGCCATTACTTCCAGCATTTCCATGCGCCGGGCTACGACTTCGCGAGCATGCCGTCGGGCCACTCGATCACGGTGGGCGCGATGTTCATCGCGTTCTTCTACATCTTCCCGAAGCTGAGGTACTTCTGGTACCTGCTGATCGTGGTGTTCGCGGGCAGCAGGATCATCGTGGGCAGCCATTACCCGTCGGACGTGATCTTCGGCGTGGCGTTCGGCTGCTACTGCACGGCGTACATCTACTACTGGATGAGGAACAGGGAGATCATCTAG |

**Table S2. Gene expression after 4 h of OMV treatment measured by real time PCR using Human Innate & Adaptive Immune Responses RT² Profiler PCR Arrays**

| **Symbol** | **Well** | **relative expression/*GAPDH*** | | **T-TEST (compared to control)** | |
| --- | --- | --- | --- | --- | --- |
|  |  | **Bp82** | **Bp82 *lpxE*** | **Bp82** | **Bp82 *lpxE*** |
| APCS | A01 | 0.844397101 | 1.436940767 | 0.297481033 | 0.235170895 |
| C3 | A02 | 1.197628372 | 0.946937169 | 0.351287074 | 0.435906766 |
| CASP1 | A03 | 1.469571196 | 1.846052785 | 0.137383557 | 0.031144658 |
| CCL2 | A04 | 0.785467099 | 1.651940218 | 0.256388071 | 0.155718559 |
| CCL5 | A05 | 1.528735104 | 7.035027833 | 0.06886406 | 0.018061993 |
| CCR4 | A06 | 9.981803856 | 1.436940767 | 0.215704132 | 0.235170895 |
| CCR5 | A07 | 2.558526869 | 2.595257971 | 0.312332534 | 0.287217888 |
| CCR6 | A08 | 5.62785415 | 1.436940767 | 0.220897503 | 0.235170895 |
| CCR8 | A09 | 0.844397101 | 1.436940767 | 0.297481033 | 0.235170895 |
| CD14 | A10 | 0.625016812 | 0.4369804 | 0.136178003 | 0.077703757 |
| CD4 | A11 | 2.194647345 | 1.687516115 | 0.058739656 | 0.057888528 |
| CD40 | A12 | 2.893546942 | 8.024915053 | 0.084527645 | 0.002053598 |
| CD40LG | B01 | 1.291987024 | 0.392151868 | 0.451028922 | 0.213913739 |
| CD80 | B02 | 1.777794591 | 30.54761551 | 0.350214402 | 0.001296315 |
| CD86 | B03 | 1.747321404 | 3.48369249 | 0.145726676 | 0.012574472 |
| CD8A | B04 | 0.129531836 | 1.063991011 | 0.010637206 | 0.475851419 |
| CRP | B05 | 0.844397101 | 1.436940767 | 0.297481033 | 0.235170895 |
| CSF2 | B06 | 49.94930397 | 1370.488592 | 0.001084924 | 0.001098285 |
| CXCL10 | B07 | 36.25366039 | 2216.881051 | 0.08305604 | 0.065186354 |
| CXCR3 | B08 | 1.353943846 | 1.09053802 | 0.440050529 | 0.479885919 |
| DDX58 | B09 | 1.628384301 | 10.55601941 | 0.14026195 | 0.002625926 |
| FASLG | B10 | 0.844397101 | 1.436940767 | 0.297481033 | 0.235170895 |
| FOXP3 | B11 | 4.547377395 | 2.512292049 | 0.274453413 | 0.332949718 |
| GATA3 | B12 | 0.844397101 | 1.436940767 | 0.297481033 | 0.235170895 |
| HLA-A | C01 | 1.884678806 | 1.513699243 | 0.051910313 | 0.04458952 |
| HLA-E | C02 | 2.165685643 | 1.693103614 | 0.105421135 | 0.013007428 |
| ICAM1 | C03 | 2.843646 | 4.132798321 | 0.00798096 | 0.010603841 |
| IFNA1 | C04 | 0.844397101 | 1.436940767 | 0.297481033 | 0.235170895 |
| IFNAR1 | C05 | 2.206269788 | 1.723971079 | 0.030313476 | 0.023317515 |
| IFNB1 | C06 | 1.675349114 | 64.78193547 | 0.109608835 | 0.043202279 |
| IFNG | C07 | 0.844397101 | 1.436940767 | 0.297481033 | 0.235170895 |
| IFNGR1 | C08 | 2.004483269 | 1.141729968 | 0.070829383 | 0.097109537 |
| IL10 | C09 | 1.587544845 | 48.03871857 | 0.087634537 | 0.019281469 |
| IL13 | C10 | 3.614167663 | 4.254841391 | 0.246535532 | 0.211443025 |
| IL17A | C11 | 0.844397101 | 1.436940767 | 0.297481033 | 0.235170895 |
| IL18 | C12 | 1.61573441 | 2.038262262 | 0.109145324 | 0.012415605 |
| IL1A | D01 | 3.088492127 | 9.178004226 | 0.018315861 | 0.006522962 |
| IL1B | D02 | 2.018670333 | 2.184699706 | 0.01015272 | 0.008894699 |
| IL1R1 | D03 | 1.369742575 | 1.964987336 | 0.118586518 | 0.001954011 |
| IL2 | D04 | 0.844397101 | 1.436940767 | 0.297481033 | 0.235170895 |
| IL23A | D05 | 3.807573688 | 66.95631252 | 0.029430497 | 0.002407019 |
| IL4 | D06 | 0.844397101 | 1.436940767 | 0.297481033 | 0.235170895 |
| IL5 | D07 | 0.222952355 | 0.545826092 | 0.189168862 | 0.243393434 |
| IL6 | D08 | 21.54681176 | 438.7399027 | 0.006313914 | 0.00165025 |
| CXCL8 | D09 | 2.211008931 | 4.744439365 | 0.055372141 | 0.001128009 |
| IRAK1 | D10 | 1.760918098 | 1.405314059 | 0.196492095 | 0.109703895 |
| IRF3 | D11 | 1.631447822 | 1.446762986 | 0.173694837 | 0.04172396 |
| IRF7 | D12 | 1.685126494 | 6.865344472 | 0.068002846 | 0.000802526 |
| ITGAM | E01 | 1.675517991 | 1.321517625 | 0.124527763 | 0.016698201 |
| JAK2 | E02 | 2.627052788 | 3.107165928 | 0.023418214 | 0.000738617 |
| LY96 | E03 | 2.045910159 | 1.698151362 | 0.030863051 | 0.069391776 |
| LYZ | E04 | 1.960019547 | 1.231503955 | 0.01761728 | 0.146578883 |
| MAPK1 | E05 | 1.605515793 | 1.10454116 | 0.122616872 | 0.211171822 |
| MAPK8 | E06 | 1.830993837 | 1.434829457 | 0.145958054 | 0.140287098 |
| MBL2 | E07 | 0.942107986 | 2.210739206 | 0.299528147 | 0.492725737 |
| MPO | E08 | 0.66851963 | 1.838179143 | 0.30766759 | 0.241422673 |
| MX1 | E09 | 0.906524658 | 52.60905229 | 0.448275238 | 0.000671814 |
| MYD88 | E10 | 1.77345231 | 2.252405492 | 0.125579036 | 0.004313729 |
| NFKB1 | E11 | 3.105035112 | 3.56672185 | 0.121209617 | 0.018571472 |
| NFKBIA | E12 | 4.250662188 | 7.304610437 | 0.000917075 | 0.011250951 |
| NLRP3 | F01 | 4.722243432 | 4.057424539 | 0.015161946 | 0.012878362 |
| NOD1 | F02 | 1.994867956 | 1.524055307 | 0.086253667 | 0.186004073 |
| NOD2 | F03 | 0.798471651 | 0.503928382 | 0.295591346 | 0.175914534 |
| RAG1 | F04 | 3.412246626 | 1.688344918 | 0.12532604 | 0.294535052 |
| RORC | F05 | 10.36728745 | 1.436940767 | 0.215519803 | 0.235170895 |
| SLC11A1 | F06 | 2.264519539 | 1.465936615 | 0.122995977 | 0.129067543 |
| STAT1 | F07 | 1.829875328 | 2.597543472 | 0.087824992 | 0.007590118 |
| STAT3 | F08 | 1.951042628 | 1.53386379 | 0.139475727 | 0.02864719 |
| STAT4 | F09 | 4.949627136 | 35.94446553 | 0.025759142 | 0.000124834 |
| STAT6 | F10 | 1.454486027 | 1.41428675 | 0.293283632 | 0.035462123 |
| TBX21 | F11 | 1.457339877 | 0.527379341 | 0.064843688 | 0.051524082 |
| TICAM1 | F12 | 1.2119809 | 2.192807414 | 0.155571323 | 0.03023155 |
| TLR1 | G01 | 2.377300209 | 0.710336644 | 0.130479095 | 0.043429306 |
| TLR2 | G02 | 2.238145772 | 1.352063359 | 0.026402245 | 0.050588207 |
| TLR3 | G03 | 0.183188948 | 0.214553141 | 0.027377498 | 0.031372429 |
| TLR4 | G04 | 1.442714946 | 0.811402453 | 0.301541836 | 0.05690057 |
| TLR5 | G05 | 0.230939686 | 11.85574062 | 0.18740509 | 0.142617284 |
| TLR6 | G06 | 1.377004461 | 0.588241738 | 0.203495762 | 0.105929008 |
| TLR7 | G07 | 2.177234729 | 7.648200746 | 0.465093219 | 0.059298349 |
| TLR8 | G08 | 45.29639665 | 54.41003633 | 0.071724988 | 0.044568026 |
| TLR9 | G09 | 16.60661705 | 8.745985551 | 0.109729787 | 0.234734658 |
| TNF | G10 | 10.07827873 | 59.281684 | 0.002810958 | 0.001062371 |
| TRAF6 | G11 | 1.313012996 | 1.25769781 | 0.288190529 | 0.057568522 |
| TYK2 | G12 | 1.935189595 | 1.694930495 | 0.143629604 | 0.042579919 |
| ACTB | H01 | 1.47085573 | 1.356851874 | 0.13083783 | 0.068854207 |
| B2M | H02 | 1.471961624 | 1.938850266 | 0.120445516 | 0.028474965 |
| GAPDH | H03 | 1 | 1 |  |  |
| HPRT1 | H04 | 1.710137403 | 1.883855959 | 0.115487472 | 0.016241532 |
| RPLP0 | H05 | 1.282407522 | 1.530257222 | 0.275331246 | 0.123490138 |

**Fig. S1. Gene expression upon LPS treatment in human THP-1-derived macrophages.** Expression of (A) *TNF*, (B) *IL10* and (C) *IFNG* was quantified by real time PCR upon treatment of LPS from uninduced Bp82 *lpxE* (UI), induced Bp82 *lpxE* (I), uninduced Bp82 Δ*lpxO* Δ*pagL lpxE* (UI), and induced Bp82 Δ*lpxO* Δ*pagL lpxE* (I). *GAPDH* was used as an internal normalization control. Statistical significance was calculated by comparison of gene expression of each pure LPS treatment to WT Bp82 LPS treated using Student’s t-test, (* *p* ≤ 0.05, ** *p* ≤ 0.01, *** *p* ≤ 0.001, **** *p* ≤ 0.0001). Experiments were carried out in biological triplicate.

**Fig. S2. Comparison of gene expression at 4 h post treatment with LPS and OMV.** Expression of (A) *TNF*, (B) *IL10* and (C) *IFNG* from groups treated with LPS or OMV from the WT Bp82, induced Bp82 *lpxE* (I) and induced Bp82 Δ*lpxO* Δ*pagL lpxE* (I). The findings indicate that both pure LPS and OMV from mutant strains expressing monophosphorylated lipid A significantly upregulated gene expression of *TNF* and *IFNG*, compared to WT Bp82 LPS and OMV treated group and untreated control group. Interestingly, there was no difference in expression of *IL10* between WT Bp82 LPS and *lpxE*-expressing LPS treated groups, while a significant difference was observed in the OMV treatment, suggesting that other components in OMVs such as proteins might drive this anti-inflammatory response. Statistical significance was calculated by comparing gene expression of each pure LPS or OMV treatment to WT Bp82 LPS or WT Bp82 OMV treated using Student’s t-test, (* *p* ≤ 0.05, ** *p* ≤ 0.01, *** *p* ≤ 0.001, **** *p* ≤ 0.0001).
